# *In Vitro* Evaluation of Skin-Related Physicochemical Properties and Biological Activities of Astaxanthin Isomers

**DOI:** 10.1021/acsomega.2c08173

**Published:** 2023-05-23

**Authors:** Masaki Honda, Yasuhiro Nishida

**Affiliations:** † Faculty of Science & Technology, Meijo University, Shiogamaguchi, Tempaku-ku, Nagoya, Aichi 468-8502, Japan; ‡ Fuji Chemical Industries, Co., Ltd., Yokohoonji, Kamiich-machi, Nakaniikawa-gun, Toyama 930-0405, Japan

## Abstract

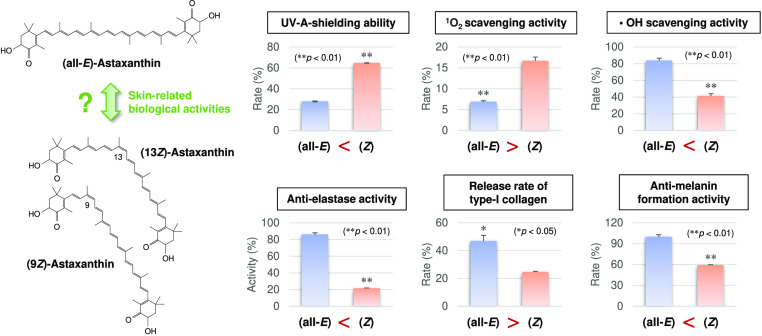

Dietary astaxanthin exists predominantly as the all-*E*-isomer; however, certain amounts of the *Z*-isomers are universally present in the skin, whose roles remain largely unknown. The aim of this study was to investigate the effects of the astaxanthin *E*/*Z*-isomer ratio on skin-related physicochemical properties and biological activities using human dermal fibroblasts and B16 mouse melanoma cells. We revealed that astaxanthin enriched in *Z*-isomers (total *Z*-isomer ratio = 86.6%) exhibited greater UV-light-shielding ability and skin antiaging and skin-whitening activities, such as anti-elastase and anti-melanin formation activities, than the all-*E*-isomer-rich astaxanthin (total *Z*-isomer ratio = 3.3%). On the other hand, the all-*E*-isomer was superior to the *Z*-isomers in singlet oxygen scavenging/quenching activity, and the *Z*-isomers inhibited type I collagen release into the culture medium in a dose-dependent manner. Our findings help clarify the roles of astaxanthin *Z*-isomers in the skin and would help in the development of novel skin health-promoting food ingredients.

## Introduction

Astaxanthin (3,3′-dihydroxy-β,β′-carotene-4,4′-dione; C_40_H_52_O_4_) is a carotenoid, belonging to the xanthophyll group, responsible for the red color of some microorganisms and seafoods.^1,2^ Astaxanthin exhibits potent antioxidant activity which is ∼100-fold higher than that of a-tocopherol, and its daily consumption is associated with a lower risk of several diseases including several cancers and eye diseases.^3−5^ Moreover, numerous studies have demonstrated that astaxanthin inhibits UV-induced skin damage and exerts positive effects on skin health.^6−9^ Astaxanthin effectively suppressed free radical-induced cell damage and matrix metalloproteinase (MMP)-1 induction in the skin after UV light irradiation *in vitro*.^8^ Furthermore, several clinical trials have demonstrated that daily astaxanthin supplementation improves indicators of crow’s feet wrinkles, elasticity, and transepidermal water loss of the skin.^6,9^ Thus, the demand for astaxanthin supplements for skin care has steadily increased in recent years.

Orally ingested carotenoids including astaxanthin are known to accumulate in the skin.^10,11^ Interestingly, it has been reported that even when (all-*E*)-carotenoids, which is the predominant carotenoid isomer in foods and supplements, are ingested, the *Z*-isomers accumulate in abundance in the skin.^12−14^ This is thought to be owing to the fact that carotenoid *Z*-isomerization might undergo during the absorption and/or carotenoid *Z*-isomers are more bioavailable than the all-*E*-isomers.^12−15^ Additionally, consumption of foods rich in carotenoid *Z*-isomers further increases the *Z*-isomer ratio in the skin.^12−14^ However, the role (i.e., significance and function) of astaxanthin *Z*-isomers in the skin remains unclear. Several studies have reported that astaxanthin *Z*-isomers show greater antioxidant and anti-inflammatory activities than those of the all-*E*-isomer.^16,17^ Moreover, we recently demonstrated that an oral diet enriched in astaxanthin *Z*-isomers (total *Z*-isomer ratio = 84.4%) increased the *Z*-isomer ratio in the skin, which inhibited UV-light-induced skin damage in guinea pigs compared to those fed a diet rich in the all-*E*-isomer (total *Z*-isomer ratio = 3.2%).^14^ Thus, astaxanthin *Z*-isomers have beneficial effects on skin health maintenance.

The purpose of this study was to investigate the effects of the *E*/*Z*-isomer ratio of astaxanthin on skin-related physicochemical properties and biological activities. Regarding the physicochemical properties, UV-A- and UV-B-shielding abilities and antioxidant activities (i.e., singlet oxygen scavenging, 2,2-diphenyl-1-picrylhydrazyl [DPPH] radical scavenging, hydroxyl radical scavenging, and superoxide anion scavenging activities) were evaluated. As to the skin-related biological activities, skin antiaging activities (i.e., anti-elastase activity and proliferation-, hyaluronic acid production-, and type I collagen production-promoting effects of human dermal fibroblasts) and skin-whitening actions (i.e., anti-tyrosinase and anti-melanin formation activities and inhibitory activity of melanin precursor [DHICA, 5,6-dihydroxyindole-2-carboxylic acid] darkening) were assessed using human dermal fibroblasts and B16 mouse melanoma cells. With some exceptions (i.e., UV-shielding abilities and antioxidant activities),^14,16^ this is the first time, to our knowledge, that these skin-related evaluations were conducted. *Z*-Isomer-rich astaxanthin was prepared from the all-*E*-isomer by thermal treatment and solid–liquid separation.^12,14^ An accurate understanding of the physicochemical properties and skin-related biological activities of astaxanthin isomers would contribute to their significance in the skin and to the development of food ingredients that promote skin health.

## Materials and Methods

### Preparation of *Z*-Isomer-Enriched Astaxanthin

*Z*-Isomer-enriched astaxanthin was prepared from the all-*E*-isomer-rich astaxanthin (synthetic astaxanthin standard; FUJIFILM Wako Pure Chemical Corp., Osaka, Japan) using thermal treatment and solid–liquid separation as previously described.^12,14^ The all-*E*-isomer-rich astaxanthin standard was dissolved in dichloromethane (CH_2_Cl_2_) at a concentration of 1 mg/mL and kept at 80 °C for 5 h. Subsequently, CH_2_Cl_2_ was evaporated under reduced pressure at 40 °C, and the resulting solid was suspended in ethanol at a concentration of 10 mg/mL with ultrasonic treatment. The suspension was left undisturbed at 4 °C for 1 h, and the insoluble substances, that is, (all-*E*)-astaxanthin crystals, were removed using a PTFE filter (0.22 μm pore size; Osaka Chemical Co., Ltd., Osaka, Japan). Lastly, ethanol was evaporated from the filtrate under reduced pressure at 40 °C, and *Z*-isomer-rich astaxanthin was obtained.

### HPLC Analysis

Astaxanthin isomers were analyzed using normal-phase HPLC with a photodiode array detector (SPD-M20A; Shimadzu, Kyoto, Japan) as previously described.^18^ Briefly, two silica gel columns connected in tandem (Luna 5 μm Silica (2), 2 mm × 150 mm × 4.6 mm, 100 Å, Phenomenex Inc., Torrance, CA) were used as the stationary phase, and a mixture of hexane, ethyl acetate, and acetone (70:20:10, v/v/v) was used as the mobile phase. The column temperature was adjusted to 40 °C, and the flow rate was maintained at 1.2 mL/min. The quantification of astaxanthin isomers was carried out by peak area integration at 470 nm. The peaks of astaxanthin *E*/*Z*-isomers in the chromatograms were identified by retention times and spectral data (i.e., whole band shapes of the absorption spectra, maximum absorption wavelengths, and relative intensities of the *Z*-peak to the absorption maximum peak of the isomer [*Q*-ratios]).^12,14,18,19^ The total (or each) *Z*-isomer ratio of astaxanthin was determined as follows
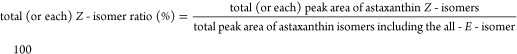


### Evaluation of UV-Light-Shielding Ability

The shielding abilities of UV-A (320–400 nm) and UV-B (280–320 nm) lights of all-*E*- and *Z*-isomer-rich astaxanthin were measured using a UV–vis spectrophotometer (V-750; Jasco Corp., Tokyo, Japan) equipped with a UV shield factor calculation program (VWSE-798; Jasco Corp., Tokyo, Japan).^14^ The astaxanthin samples were dissolved in ethyl acetate at concentrations of 1–20 μM based on a previous work of our research group.^20^

### Evaluation of Antioxidant Activities

The antioxidant activities of the all-*E*- and *Z*-isomer-rich astaxanthin were determined using four different assays for evaluating the singlet oxygen, DPPH radical, hydroxyl radical, and superoxide anion scavenging activities. The singlet oxygen scavenging activity was determined using an antioxidant capacity assay kit for singlet oxygen (SL2010; SakuLab Science, Inc., Kanagawa, Japan) and was performed by SakuLab Science, Inc. (Kanagawa, Japan).^20^ The evaluations on DPPH radical, hydroxyl radical, and superoxide anion scavenging activities were done according to previously described methods^20−23^ and were performed by Kirei Testing Labo Co., Ltd. (Tokyo, Japan). The astaxanthin samples were diluted by chloroform for the assay of the singlet oxygen scavenging activity and diluted by 0.5% dimethyl sulfoxide (DMSO) solution for the assays of the other three antioxidant activities at concentrations of 1–50 μM.

### Evaluation of Skin Antiaging Activities

The differences in the skin antiaging activities of all-*E*- and *Z*-isomer-rich astaxanthin were investigated by evaluating their anti-elastase activity and proliferation-, hyaluronic acid production-, and type I collagen production-promoting effects in human dermal fibroblasts according to the previously described procedures.^20,24−27^ The astaxanthin samples were diluted by 0.5% DMSO solution for the assay of the anti-elastase activity at concentrations of 1–50 μM and for the assays of the other three skin antiaging activities at concentrations of 1–10 μM. These *in vitro* evaluations were performed by Kirei Testing Labo Co., Ltd. (Tokyo, Japan).

### Evaluation of Skin-Whitening Action

The effects of astaxanthin with different *Z*-isomer ratios on skin-whitening actions were investigated. Specifically, anti-tyrosinase and anti-melanin formation activities as well as the inhibitor activity for melanin precursor (DHICA) darkening were evaluated according to the previously described procedures.^20,28−32^ In the evaluation of the anti-melanin formation activity, B16 mouse melanoma cells were used.^29,30^ The astaxanthin samples were diluted by 0.5% DMSO solution for the assay of the anti-tyrosinase activity at concentrations of 1–50 μM, diluted by the culture medium for evaluating the anti-melanin formation activity at concentrations of 1–20 μM, and diluted by ultrapure water for evaluating the inhibitor activity for melanin precursor (DHICA) darkening at concentrations of 1–20 μM. These *in vitro* evaluations were performed by Kirei Testing Labo Co., Ltd. (Tokyo, Japan).

### Statistical Analysis

All data were collected in triplicates and expressed as the mean ± standard deviation. The differences in mean values were analyzed by Welch’s *t*-test using EZR software (version 1.55; Saitama Medical Center, Jichi Medical University, Saitama, Japan), and significance was set at *p* < 0.01 and *p* < 0.05.

## Results

### Profile of Astaxanthin Isomers

**Figure 1:**
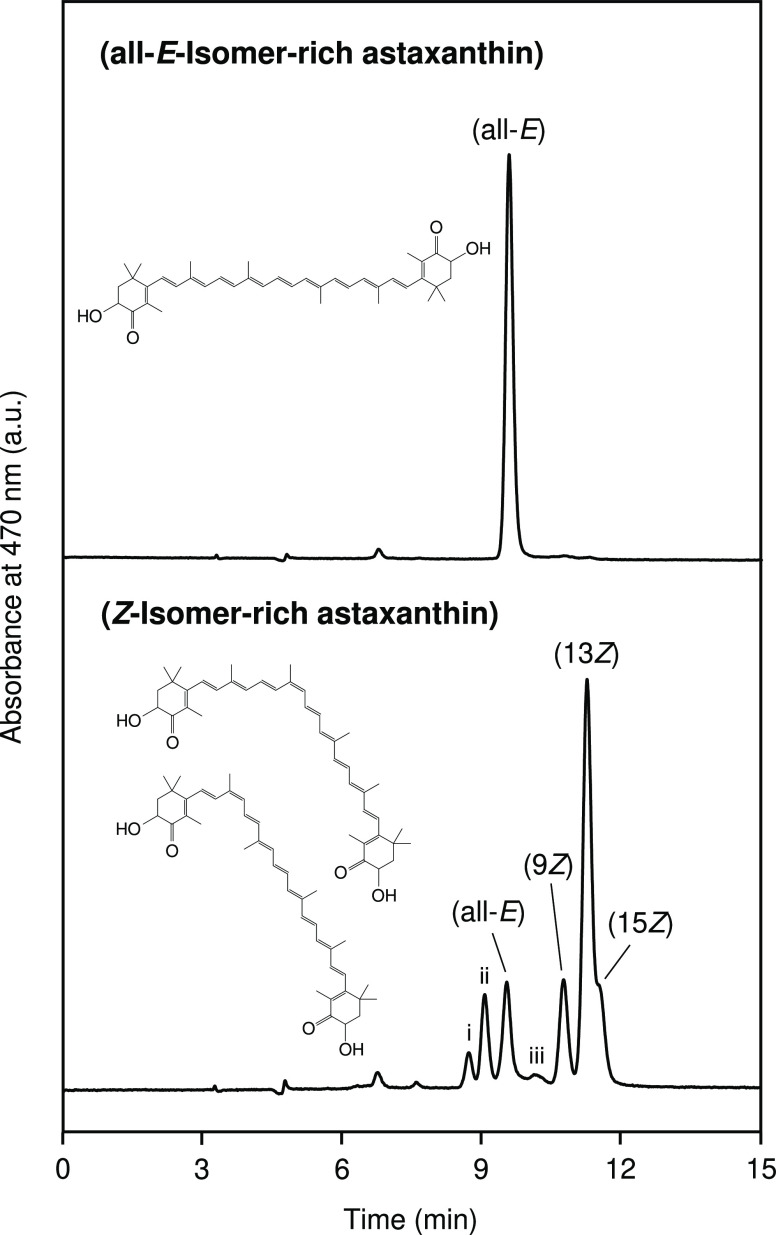
Normal-phase HPLC chromatograms of all-*E*-isomer-rich astaxanthin (total *Z*-isomer-ratio = 3.3%) and *Z*-isomer-rich astaxanthin (total *Z*-isomer-ratio = 86.6%). Labels of (all-*E*), (9*Z*), (13*Z*), and (15*Z*) denote (all-*E*)-, (9*Z*)-, (13*Z*)-, and (15*Z*)-astaxanthin, respectively, and peaks of i, ii, and iii are assigned to unknown astaxanthin *Z*-isomers (Table 1).

The chromatograms of the astaxanthin standard and treated astaxanthin are shown in Figure 1. The astaxanthin standard consisted mostly of the all-*E*-isomer (total *Z*-isomer ratio = 3.3%), while the total *Z*-isomer ratio of the treated astaxanthin increased by 86.6%. Six different *Z*-isomers were detected, including the 9*Z*-, 13*Z*-, and 15*Z*-isomers and three unknown *Z*-isomers (perhaps the di-*Z*-isomers) (Table 1). The 13*Z*-isomer exhibited the highest percentage (48.4%), followed by the 9*Z*-isomer (12.4%) and the 15*Z*-isomer (9.9%). The astaxanthin standard and treated astaxanthin were used as the all-*E*- and *Z*-isomer-rich astaxanthin, respectively, and they were used for the *in vitro* evaluations of skin-related physicochemical properties and biological activities.

**Table 1:** Absorption Maxima (λ_max_) and Relative Intensities of the *Z*-Peaks (*Q*-Ratio) for Geometrical Astaxanthin Isomers Separated and Observed Using Normal-Phase HPLC^a^

		λ_max_ (nm)		*Q*-ratio	
label	isomer^b^	observed	reported^b^	observed	reported^b^
i	(*xZ*)-astaxanthin	457	–	ND	–
ii	(*xZ*)-astaxanthin	457	–	ND	–
(all-*E*)	(all-*E*)-astaxanthin	474	472	ND	–
iii	(*xZ*)-astaxanthin	462	–	ND	–
(9*Z*)	(9*Z*)-astaxanthin	367, 467	365, 465	0.22	0.20
(13*Z*)	(13*Z*)-astaxanthin	366, 464	366, 465	0.49	0.52
(15*Z*)	(15*Z*)-astaxanthin	366, 467	365, 468	0.54	0.56

a Values and peak designations are obtained from the chromatograms in Figure 1. −, not assigned. ND, not detected substantially.

b Tentatively assigned in the literature.^12,14,18,19^

### UV-Light-Shielding Ability of Astaxanthin Isomers

**Figure 2:**
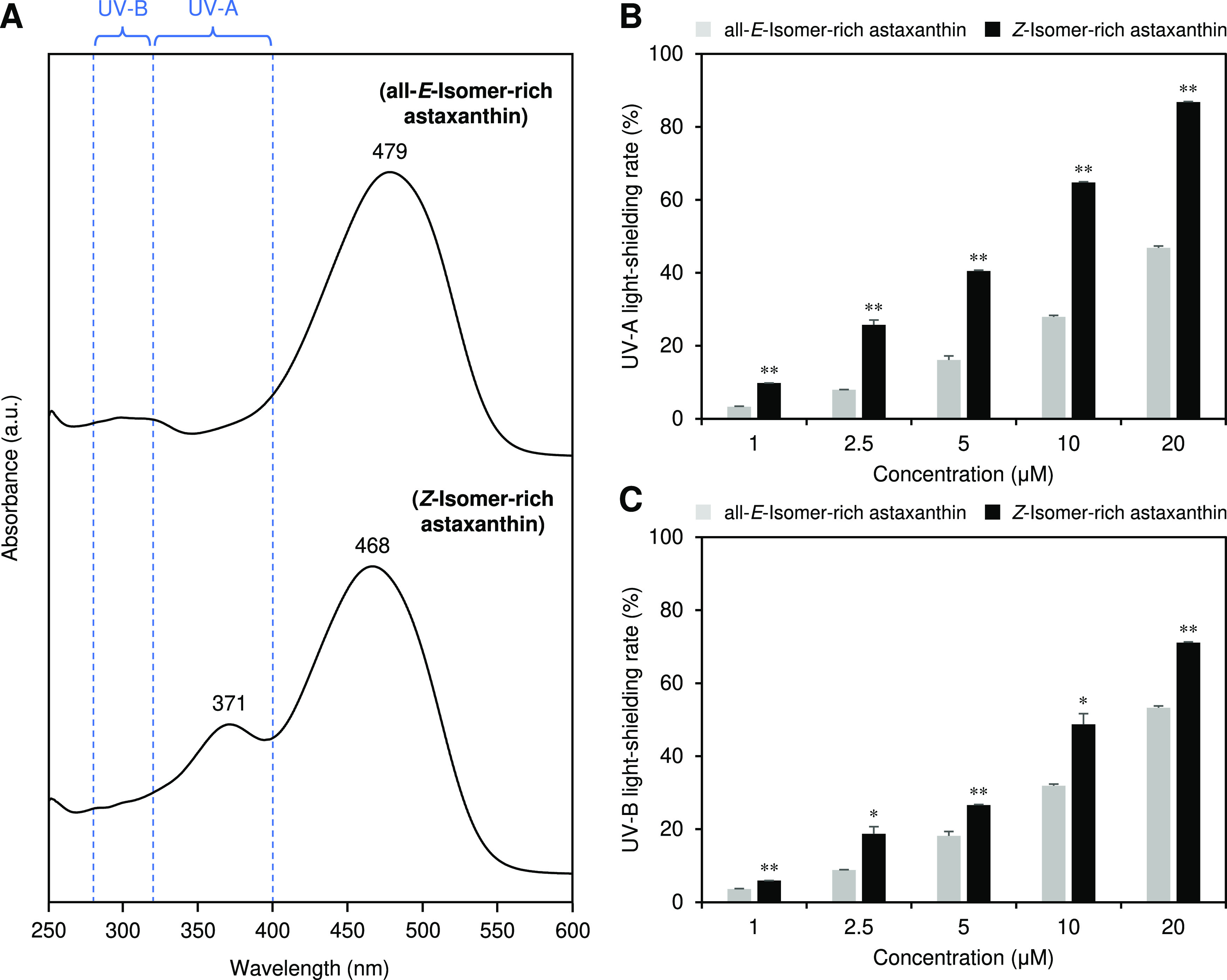
(A) Absorption spectra and (B) UV-A- and (C) UV-B-shielding abilities of all-*E*- and *Z*-isomer-rich astaxanthin. UV-A and UV-B indicate ranges of 320–400 and 280–320 nm, respectively. The error bars show standard deviations across three independent tests. Asterisks (*) indicate a statistically significant difference between all-*E*- and *Z*-isomer-rich astaxanthin (*p < 0.05, **p < 0.01, Welch’s *t*-test).

The UV-A and UV-B-shielding abilities of all-*E*- and *Z*-isomer-rich astaxanthin were evaluated based on their absorption wavelengths (Figure 2A). The *Z*-isomer-enriched astaxanthin had a shorter absorption spectrum than that of the all-*E*-isomer-rich astaxanthin. The maximum absorption wavelengths specific to the all-*E*- and *Z*-isomer-rich astaxanthin were 479 and 468 nm, respectively, and the *Z*-isomer-rich astaxanthin had a characteristic maximum absorption wavelength at 371 nm. The *Z*-isomer-rich astaxanthin exhibited 2- and 1.5-times greater UV-A- and UV-B-shielding abilities, respectively, than those of the all-*E*-isomer-rich astaxanthin (Figure 2B,D).

### Antioxidant Activities of Astaxanthin Isomers

**Figure 3:**
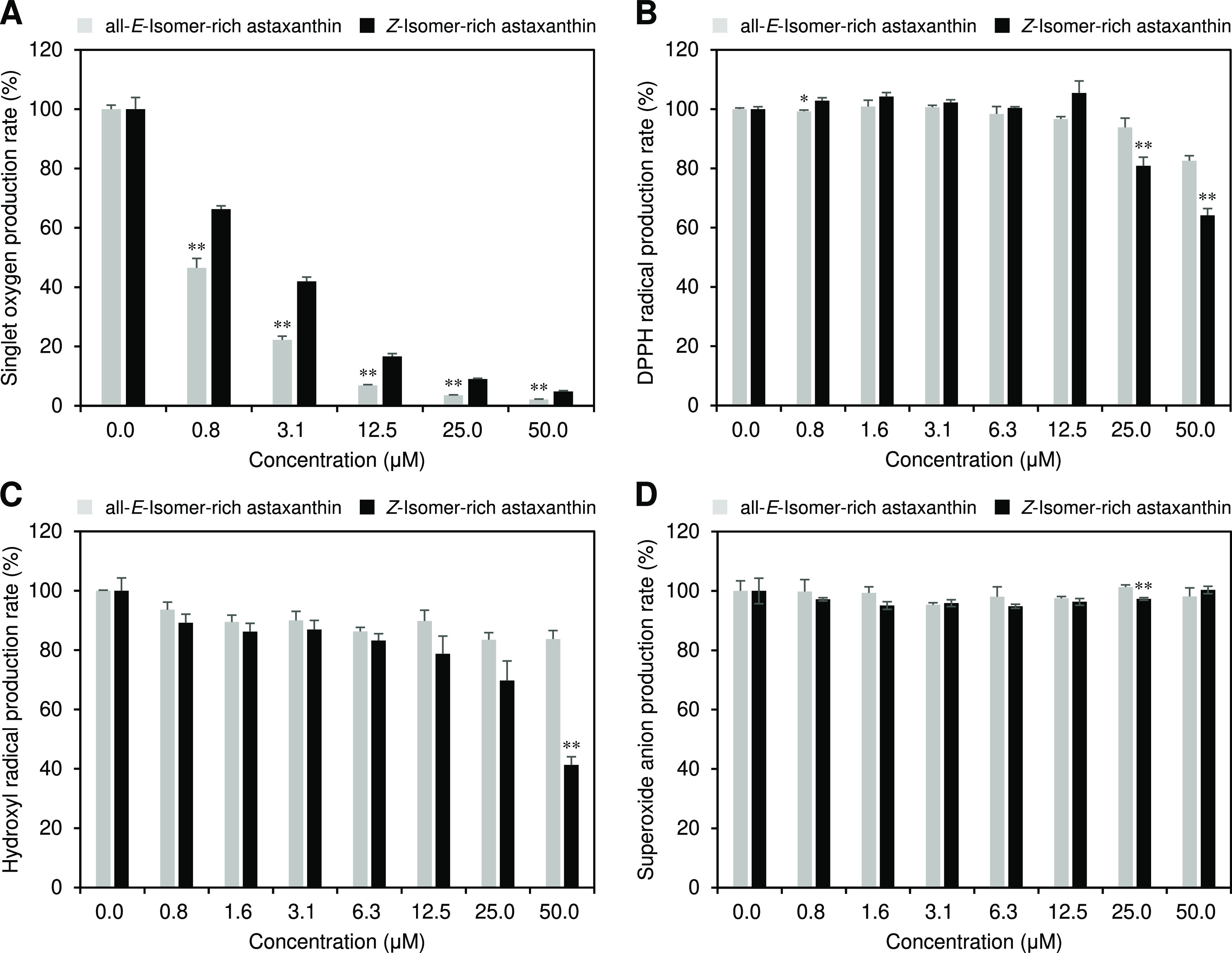
(A) Singlet oxygen scavenging, (B) DPPH radical scavenging, (C) hydroxyl radical scavenging, and (D) superoxide anion scavenging activities of all-*E*- and *Z*-isomer-rich astaxanthin. The error bars show standard deviations across three independent tests. Asterisks (*) indicate a statistically significant difference between carotenoids rich in all-*E*-isomers and *Z*-isomers (*p < 0.05, **p < 0.01, Welch’s *t*-test).

We compared the antioxidant activities of all-*E*- and *Z*-isomer-rich astaxanthin by investigating their singlet oxygen, DPPH radical, hydroxyl radical, and superoxide anion scavenging activities (Figure 3). Both all-*E*- and *Z*-isomer-rich astaxanthin exhibited potent singlet oxygen activity, but the former showed superior activity (Figure 3A). The IC_50_ values of the all-*E*- and *Z*-isomer-rich samples for the singlet oxygen scavenging activity were 0.71 and 1.77 μM, respectively. On the other hand, *Z*-isomer-enriched astaxanthin exhibited greater DPPH radical and hydroxyl radical scavenging activities than all-*E*-isomer-rich astaxanthin (Figure 3B,C). Both all-*E*- and *Z*-isomer-rich astaxanthin showed little superoxide anion scavenging activity (Figure 3D).

### Skin Antiaging Activities of Astaxanthin Isomers

**Figure 4:**
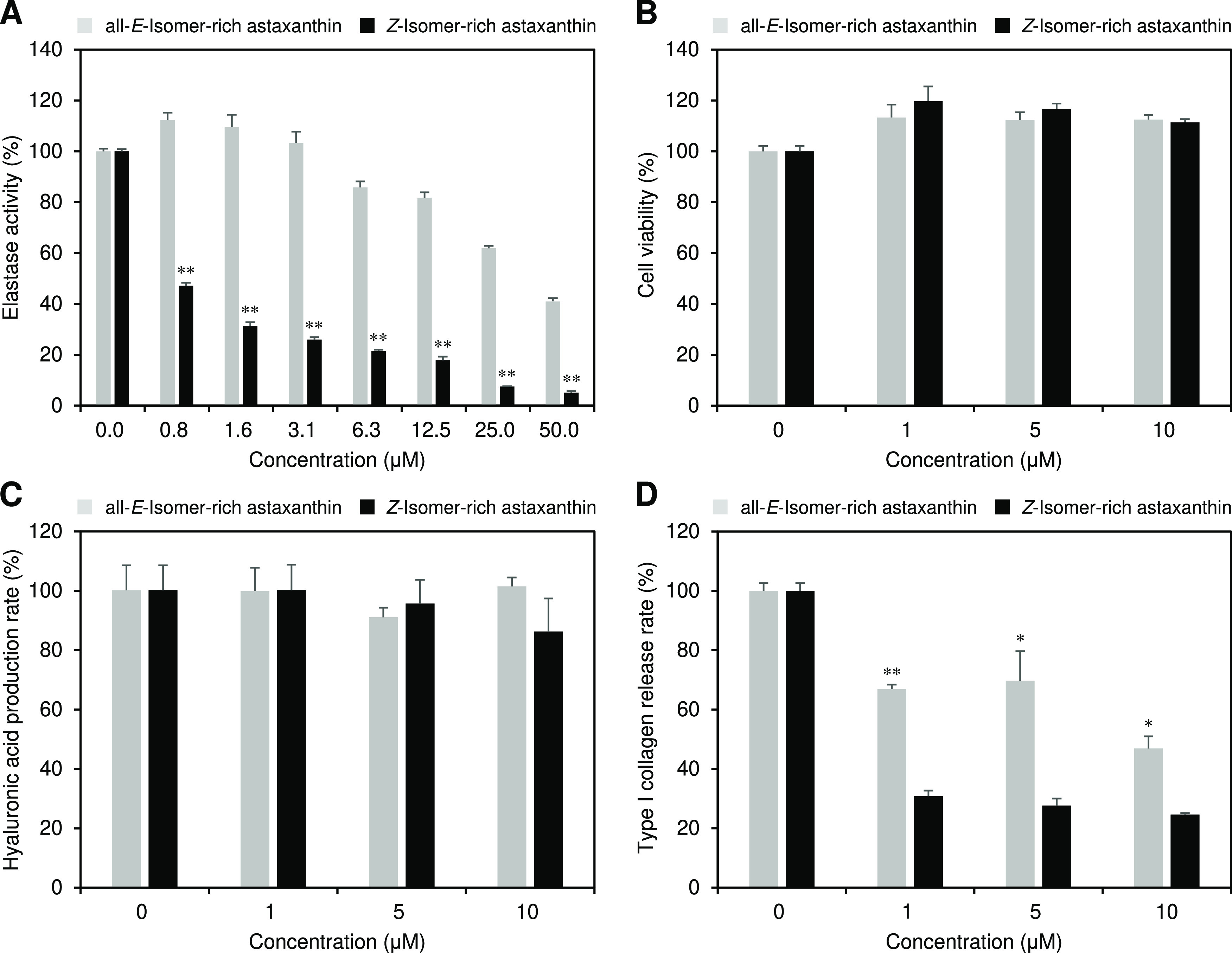
(A) Anti-elastase activity, (B) proliferation- and (C) hyaluronic acid production-promoting effects, and (D) release rate of type I collagen of all-*E*- and *Z*-isomer-rich astaxanthin. The error bars show standard deviations across three independent tests. Asterisks (*) indicate a statistically significant difference between carotenoids rich in all-*E*-isomers and *Z*-isomers (*p < 0.05, **p < 0.01, Welch’s *t*-test).

We investigated the influence of the astaxanthin *E*/*Z*-isomer ratio on anti-elastase activity and proliferation-, hyaluronic acid production-, and type I collagen production-promoting effects, which are important indicators of skin antiaging (Figure 4). Astaxanthin isomers exhibited strong anti-elastase activity, and *Z*-isomer-rich astaxanthin showed greater activity than that of all-*E*-isomer-rich astaxanthin (Figure 4A). For example, 0.8 μM *Z*-isomer-rich astaxanthin solution showed roughly equivalent activity to the 50 μM all-*E*-isomer-rich sample. The IC_50_ values of the all-*E*- and *Z*-isomer-rich astaxanthin for the anti-elastase activity were 36.3 and 0.5 μM, respectively. Both all-*E*- and *Z*-isomer-rich astaxanthin significantly promoted the proliferation of human dermal fibroblasts compared to the control solution without astaxanthin, but no differences were observed between the isomers (Figure 4B). Astaxanthin isomers had no effect on hyaluronic acid production (Figure 4C), whereas they inhibited type I collagen production, and *Z*-isomer-rich astaxanthin showed greater inhibitory effect than all-*E*-isomer-rich astaxanthin (Figure 4D). For example, the 10 μM all-*E*- and *Z*-isomer-rich astaxanthin solutions inhibited 53.1 and 75.4% of type I collagen production, respectively, compared to the control solution.

### Skin-Whitening Actions of Astaxanthin Isomers

**Figure 5:**
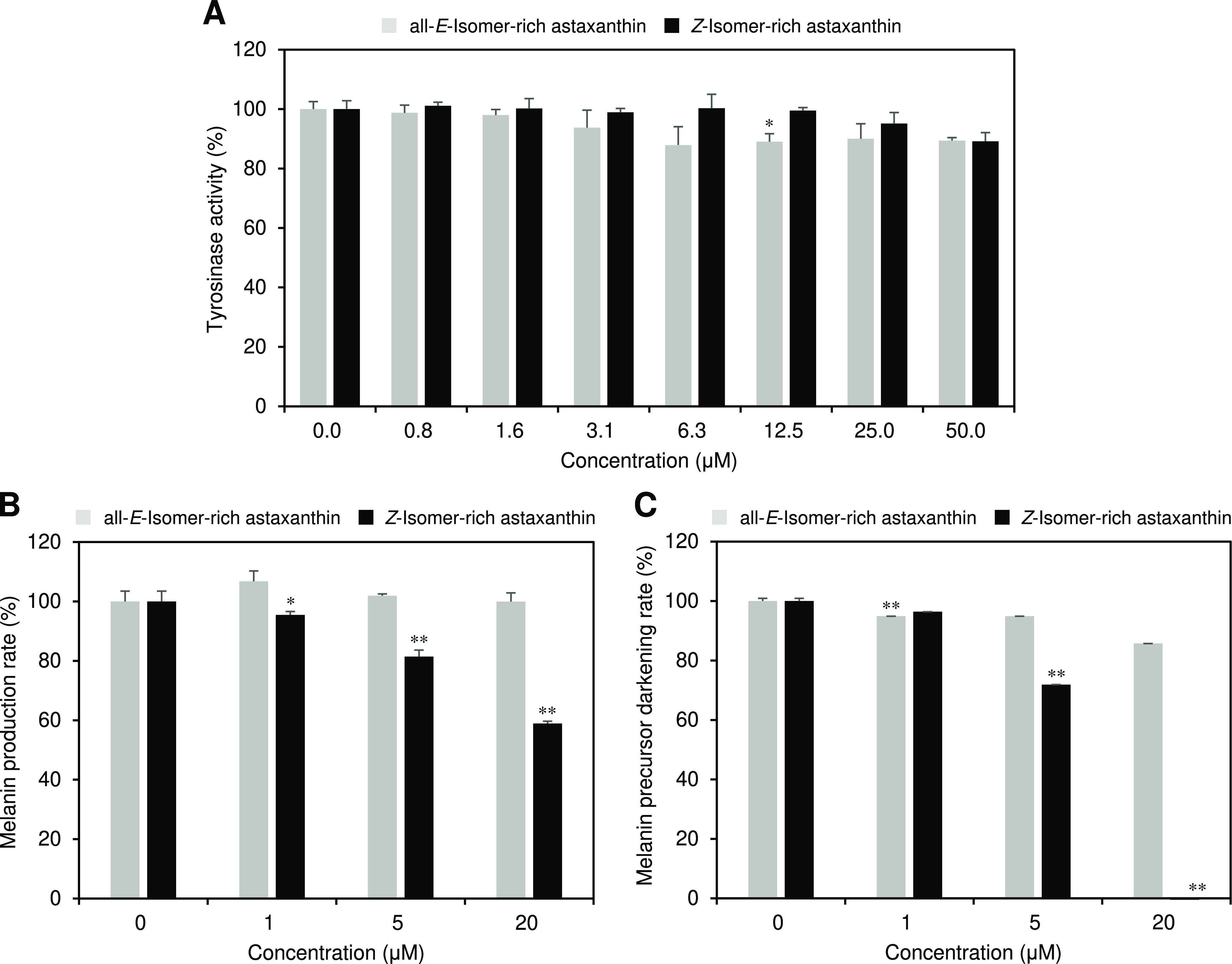
(A) Anti-tyrosinase and (B) anti-melanin formation activities and (C) inhibitor activity of melanin precursor (DHICA) darkening of all-*E*- and *Z*-isomer-rich astaxanthin. The error bars show standard deviations across three independent tests. Asterisks (*) indicate a statistically significant difference between carotenoids rich in all-*E*-isomers and *Z*-isomers (*p < 0.05, **p < 0.01, Welch’s *t*-test).

We evaluated the skin-whitening actions of astaxanthin by investigating their anti-tyrosinase and anti-melanin formation activities as well as the inhibitor activity of melanin precursor (DHICA) darkening (Figure 5). Astaxanthin isomers slightly inhibited the tyrosinase activity, but there was little difference in the activity between isomers (Figure 5A). Regarding the anti-melanin formation activity, all-*E*-isomer-rich astaxanthin did not show the activity at the concentrations tested (1–10 μM), whereas the *Z*-isomer-rich astaxanthin exhibited strong activity, e.g., 10 μM *Z*-isomer-rich astaxanthin solution inhibited 41.1% of melanin formation compared to that of the control solution without astaxanthin (Figure 5B). Furthermore, *Z*-isomer-rich astaxanthin exhibited an extremely stronger inhibitor activity of melanin precursor (DHICA) darkening than that of the all-*E*-isomer-rich astaxanthin (Figure 5C). For example, 10 μM all-*E*-isomer-rich astaxanthin solution inhibited only 14.3% of the melanin precursor darkening compared to that of the control solution without astaxanthin, whereas the *Z*-isomer-rich astaxanthin completely inhibited the activity. The IC_50_ values of the all-*E*- and *Z*-isomer-rich samples for the inhibitor activity were estimated to be ∼20 and 6.8 μM, respectively.

## Discussion

Orally ingested astaxanthin accumulates in the skin, prevents UV-induced skin damage, and improves the skin condition.^6−11^ A certain amount of astaxanthin *Z*-isomers is present in the skin even after ingestion of the all-*E*-isomer, but their roles remain unknown.^12,14^ The present study found significant differences in several skin-related physicochemical properties and biological activities between all-*E*- and *Z*-isomer-rich astaxanthin.

*Z*-Isomer-rich astaxanthin showed greater UV-A and UV-B shielding abilities than the all-*E*-isomer-rich astaxanthin (Figure 2B,C). This could be due to the absorption wavelength shift to the short wavelength side accompanying the *Z*-isomerization (Table 1). Furthermore, a characteristic maximum absorption wavelength at the UV-A region of the *Z*-isomers could also contribute to their greater UV-shielding abilities (Figure 2A). Thus, astaxanthin *Z*-isomers may better reduce UV-induced skin damage than the all-*E*-isomer. Indeed, our recent *in vivo* study showed that guinea pigs supplemented with *Z*-isomer-rich astaxanthin showed less UV-induced skin damage than those supplemented with all-*E*-isomer-rich astaxanthin.^14^ The antioxidant activity results varied depending on the assay used. Namely, all-*E*-isomer-rich astaxanthin showed greater singlet oxygen scavenging activity than the *Z*-isomer-rich astaxanthin (Figure 3A), whereas the opposite results were obtained on DPPH radical and hydroxyl radical scavenging activities (Figure 3B,C). Sakemi et al. reported that, in the other carotenoid, lycopene, the all-*E*-isomer showed higher singlet oxygen scavenging activity than the *Z*-isomers,^33^ indicating that carotenoids commonly have decreased singlet oxygen scavenging activity upon isomerization from the all-*E*- to the *Z*-isomers. Carotenoids quench singlet oxygen through a physical mechanism involving the transfer of excitation energy, followed by thermal deactivation.^34^ Accordingly, the presence of *Z*-structures in the polyene chain of carotenoid molecules might reduce the efficiency of the excitation energy transfer. Nevertheless, astaxanthin had potent singlet oxygen scavenging activity even in its *Z*-isomers (IC_50_ value = 1.77 μM). Yang et al. reported similar results on the radical scavenging activities of astaxanthin isomers, showing that several astaxanthin *Z*-isomers (i.e., the 9*Z*- and 13*Z*-isomers) exhibited greater oxygen radical absorbing capacity and DPPH radical scavenging activity.^16^ The mechanisms underlying these effects are currently unknown, but it could be due to differences in thermodynamic stability among astaxanthin *Z*-isomers. In other words, the higher energy levels of astaxanthin *Z*-isomers compared with the all-*E*-isomer may be responsible for its higher reactivity with radical species.^35,36^

Several clinical trials showed that astaxanthin on the dermal layer contributed to the improvement of wrinkles.^9,37−39^ This indicates that astaxanthin serves to maintain the extracellular matrix (ECM) of the dermis layer. In particular, it is believed that astaxanthin quenches singlet molecular oxygen,^40,41^ which is involved in the damage of collagen-based ECM-producing fibroblasts,^8^ and also inhibits enzymatic activities and/or suppresses the gene expression of MMPs,^42−48^ which are involved in the degradation of ECM. In this study, we found a direct inhibitory effect on elastase (Figure 4A). This is a novel finding regarding the effect of astaxanthin on degradation of the ECM in the dermis. In the present study, seemingly astaxanthin isomers, especially the *Z*-isomers, suppressed the production of collagen from fibroblasts (Figure 4D). It should be noted that this is because the amount of type I collagen production was evaluated based on the amount of supernatant fluid in the culture medium, and only the excretory portion of type I collagen production into the culture medium under naive conditions was evaluated. Some information on the effect of astaxanthin on collagen biosynthesis suggests that, under oxidative stress, such as UV or other light irradiation or the addition of pro-oxidants, skin fibroblasts are impaired by singlet oxygen and by the production of inflammatory cytokines.^47−49^ Although astaxanthin has been shown to exert a preventive effect on the inhibition of collagen biosynthesis by protecting against these insults, there are no reports of direct regulation of gene expression or biosynthesis promotion on naive fibroblasts. Therefore, the dose-dependent decrease in the rate of collagen released into the medium by the astaxanthin isomer may be due to the enhancement of ECM integrity by them as a result of suppression or direct inhibition and/or the gene expression of MMPs, including elastase. Therefore, the balance between type I collagen production and proteolysis by astaxanthin isomers should also be examined in the future.

We observed large differences in skin-whitening actions between all-*E*- and *Z*-isomer-rich astaxanthin. Namely, all-*E*-isomer-rich astaxanthin showed little anti-melanin formation activity and inhibitor activity of melanin precursor (DHICA) darkening, whereas *Z*-isomer-rich astaxanthin exhibited strong activities for both (Figure 5B,C). As the anti-tyrosinase activity of all-*E*- and *Z*-isomer-rich astaxanthin was nearly equivalent (Figure 5A), the skin-whitening effects of *Z*-isomer-enriched astaxanthin could be due to the regulation of related gene expression, such as tyrosinase-related proteins 1 and 2 (TRP-1 and -2) and microphthalmia-associated transcription factor (MITF) genes.^6,50,51^ In fact, Nakajima et al. reported that astaxanthin significantly suppressed expression of TRP-1 and MITF at the transcriptional and translational levels (note that the astaxanthin isomer ratio was not stated).^52^ The superior UV-light-shielding ability of astaxanthin *Z*-isomers could have contributed to the inhibitory activity of melanin precursor darkening.^32^ Further research is required to elucidate the mechanisms underlying the potent skin-whitening actions of astaxanthin *Z*-isomers, but our observations describing these effects of the *Z*-isomers are an important finding for their application in skin care supplements and cosmetics.

In conclusion, we investigated the effects of the astaxanthin *E*/*Z*-isomer ratio on skin-related physicochemical properties and biological activities and showed significant differences in their properties and activity between all-*E*- and *Z*-isomers. *Z*-Isomer-rich astaxanthin had many superior properties related to improving skin conditions compared with the all-*E*-isomer-rich astaxanthin, including UV-A- and UV-B-shielding abilities, DPPH and hydroxyl radical scavenging activities, anti-elastase activity, anti-melanin formation activity, and inhibitor activity of melanin precursor (DHICA) darkening. The facts that the *Z*-isomers have extremely high anti-elastase activity and skin-whitening actions (i.e., anti-melanin formation activity and inhibitor activity of melanin precursor darkening) are particularly valuable discoveries. Thus, as a skin condition-improving agent for use in supplements and cosmetics, the *Z*-isomers may be superior to the all-*E*-isomer. However, it should be noted that the all-*E*-isomer is superior to the *Z*-isomers in singlet oxygen scavenging activity and the *Z*-isomers may inhibit type I collagen production. These differences in skin-related biological activities between astaxanthin isomers are not correlated with their antioxidant activity, which might be associated with the regulation of specific gene expression. Even when (all-*E*)-carotenoids, which are the predominant isomer in foods, are orally ingested, *Z*-isomers are still detected in human and animal skins; however, their functions and mechanisms are unknown. The differences in skin-related physicochemical properties and biological activities between the all-*E*- and *Z*-isomers described in this study would be important in uncovering these mechanisms.
